# Co-Mutation of CREBBP/EP300 and POLE/POLD1 Identifies a TMB-High Subset of MSS CRC

**DOI:** 10.3390/ijms27157027

**Published:** 2026-08-05

**Authors:** Mariia Gusakova, Fedor Sharko, Eugenia Boulygina, Ksenia Maksimova, Maxim Patrushev

**Affiliations:** National Research Center “Kurchatov Institute”, 123182 Moscow, Russia

**Keywords:** colorectal cancer, MSS, TMB-High, CREBBP/EP300 mutations, POLE/POLD1 mutations, immunotherapy

## Abstract

Identifying immunotherapy biomarkers for colorectal cancer (CRC) beyond MSI-high status is challenging. We evaluated CREBBP/EP300 and POLE/POLD1 mutations as candidate markers using public cohorts (MSK-CHORD, *n* = 5493; TCGA, *n* = 528; MetTropism, *n* = 24,496; ICI-treated, *n* = 1610). Regression with SHAP attribution, survival analyses, and pan-cancer transcriptomic profiling (n = 7628) were performed. CREBBP/EP300 mutations were independently associated with TMB-high MSS tumors (*p* < 0.00001). The co-mutation group (CREBBP/EP300 + POLE/POLD1; CoMut) was the strongest predictor of elevated TMB level (β = 3.63; *p* < 10^−16^; exp(β) = 37.7) with a median TMB of 155.9 vs. 7.4 and 5.4 Mut/Mb in single-mutation groups. CoMut also associated with improved survival (HR = 0.57, *p* = 0.035). In the ICI-treated cohort, both single-mutation groups (CREBBP/EP300-MUT-only; POLE/POLD1-MUT-only) and showed longer overall survival than wild type (34 and 28 vs. 17 months; *p* < 0.05), but only CREBBP/EP300 mutations independently reduced mortality risk (HR = 0.71, *p* = 0.0025). All groups shared immune-inflammatory transcriptomic enrichment. These findings support CREBBP/EP300 mutations as candidate immunotherapy biomarkers, and co-mutations with POLE/POLD1 define a TMB-high MSS subgroup that could refine TMB testing, pending prospective validation.

## 1. Introduction

Colorectal cancer (CRC) remains one of the three most common cancers worldwide and a leading cause of cancer-related mortality [1]. Despite major advances in understanding CRC pathogenesis and the growing number of molecular biomarkers guiding targeted and immunotherapies, treatment options for most patients remain limited to chemotherapy combined with anti-angiogenic and/or EGFR-targeted agents [2,3,4].

CRC is characterized by a substantial proportion of patients with a hypermutator phenotype (~15% of cases are microsatellite instability-high (MSI-High)), in contrast to other gastrointestinal malignancies [5]. A recently identified, relatively rare subgroup (2–8% of cases) harbors mutations in DNA polymerases ε and δ (POLE and POLD1), with both germline and somatic mutations serving as biomarkers [5,6]. The main focus has been on mutations within the exonuclease domains (EDM) of POLE and POLD1, but a substantial number of potentially function-impairing mutations may occur outside these domains (non-EDM mutations). The clinical interpretation of both de novo EDM and non-EDM mutations remains challenging [7,8,9,10]. Accordingly, clinical guidelines recommend parallel assessment of TMB (tumor mutational burden) to confirm the presence of an ultra-hypermutated tumor genome (≥50 Mut/Mb) in POLE and POLD1 mutant cases, as this provides evidence of polymerase dysfunction [11].

The role of TMB-High as an independent predictor of treatment response and improved survival in colorectal cancer remains uncertain, despite its tumor-agnostic FDA approval for pan-cancer immunotherapy selection at TMB ≥ 10 Mut/Mb [12]. The problem of the non-universality of this biomarker, methodological requirements and interlaboratory inconsistency in the results of TMB prediction remains relevant [13,14]. Similarly, PD-L1 expression in colorectal cancer does not guide immunotherapy selection in clinical practice, in contrast to many other solid tumors [15,16].

Recent studies have highlighted the link between hypermutator phenotypes in diverse cancers and alterations in genes encoding epigenetic regulators [17]. Several in silico studies have indicated that mutations in CREBBP and EP300 are associated with genomically unstable solid tumors (MSI-High, TMB-High) and may serve as potential biomarkers for immunotherapy selection [18,19,20,21,22]. Publications have reported an association between TMB and MSI status in colorectal cancer samples and mutations in CREBBP and EP300. These genes encode the proteins CBP and p300, which function as ubiquitously co-expressed histone acetyltransferases (HATs) or lysine acetyltransferases (KATs), acting as transcriptional co-activators and modifying both histone and non-histone proteins. CBP and p300 play key roles in activating enzymes across multiple DNA repair pathways [23,24,25,26], and their dysfunction leads to destabilization of these systems. Intriguingly, evidence suggests that DNA replication is regulated through acetylation of two major endonucleases involved in Okazaki fragment processing, FEN1 and DNA2 [27,28]. There are also isolated studies investigating the direct acetylation of POLD1 by p300 [29].

Despite these mechanistic insights, colorectal tumors harboring CREBBP and EP300 mutations have not been systematically studied. In this study, we performed a comprehensive in silico analysis of CREBBP and EP300 mutations in colorectal cancer. These mutations were evaluated for their association with the hypermutator phenotype and response to immunotherapy, and were compared with POLE/POLD1-mutant tumors. We investigated co-mutations in histone acetyltransferases and DNA polymerases as a molecular subgroup associated with hypermutated MSS tumors across colorectal cancer and pan-cancer cohorts, which have not been previously described.

## 2. Results

### 2.1. Overview of Mutation Frequencies and Key Clinical and Histopathological Tumor Characteristics

The proportions of tumors exhibiting genomic instability based on standard biomarkers in the overall CRC MSK-CHORD cohort (*n* = 5493) were as follows: TMB-High, 18.2% (*n* = 999); MSI-High, 10.6% (*n* = 585); and POLE/POLD1-MUT, 9.0% *(n* = 495). Tumors harboring mutations in CREBBP and/or EP300 accounted for 10.5% of cases, including 5.0% with CREBBP-MUT, 3.6% with EP300-MUT, and 1.9% with concurrent CREBBP/EP300-MUT.

**Figure 1:**
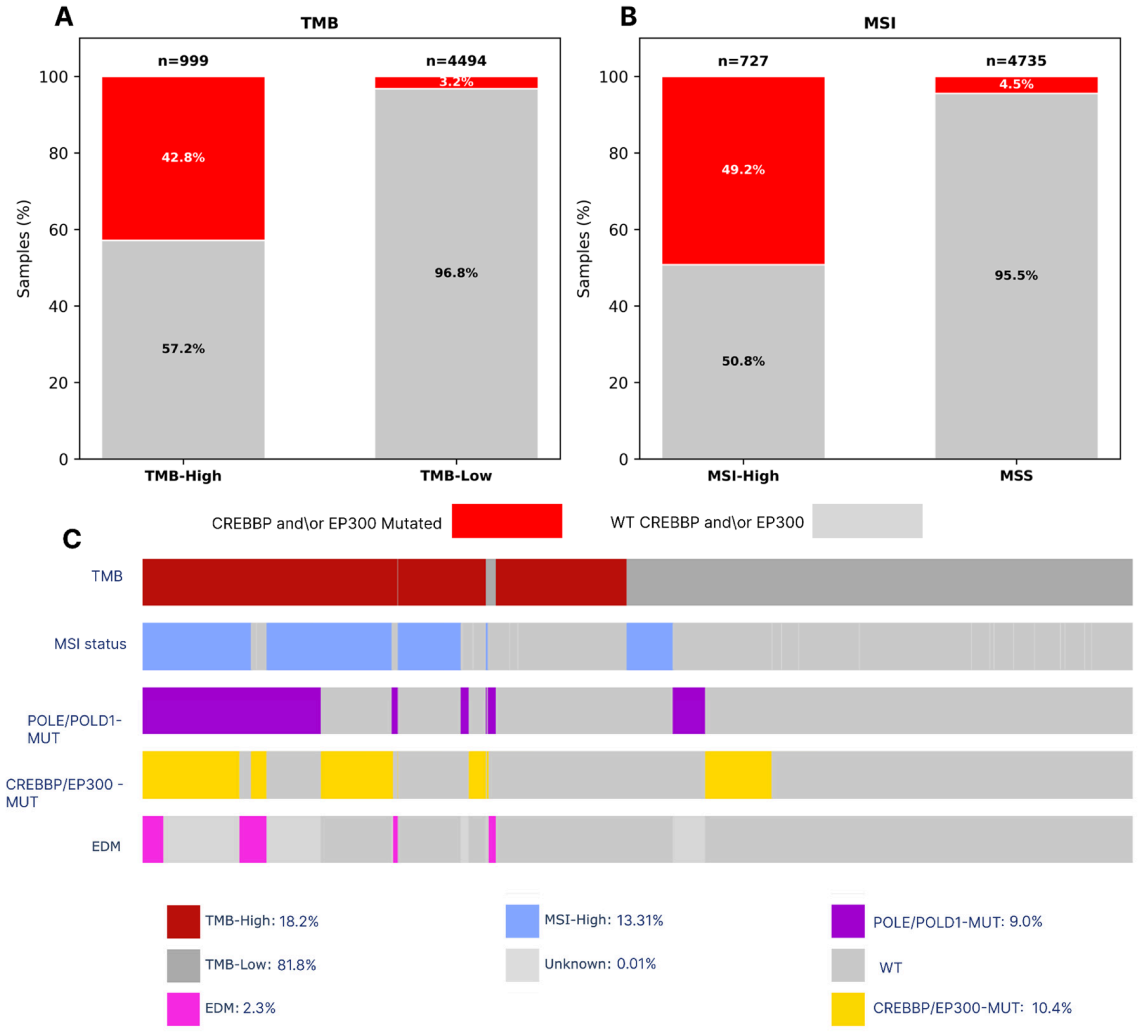
Structure of the CRC patient cohort from MSK-CHORD according to genomic instability markers (TMB, MSI status, and mutations in POLE and POLD1) and the mutational status of CREBBP and EP300. To improve clarity and readability given the large cohort size, the figure includes zoomed-in regions highlighting samples with positive biomarker statuses. (**A**) Proportion of samples harboring CREBBP and/or EP300 mutations stratified by TMB status. (**B**) Proportion of samples with CREBBP and/or EP300 mutations stratified by MSI status (MSIsensor score). (**C**) Genomic landscape of TMB, MSI, POLE/POLD1 (including EDM and non-EDM status), and CREBBP/EP300 in CRC. This plot highlights a distinct subset of tumors that are MSI-High and/or TMB-High with mutations in CREBBP and/or EP300, but without mutations in POLE/POLD1. Additionally, an isolated group of genomically stable tumors with CREBBP/EP300 mutations is apparent. The distribution of samples between genomically unstable and stable tumors is similar for POLE/POLD1-MUT and CREBBP/EP300-MUT. The proportions shown in the legend are calculated relative to the total cohort for each respective marker.

Mutations in the target genes were observed across all genomically unstable subgroups: TMB-High in 42.8% CREBBP/EP300-MUT cases vs. 3.2% WT. Mutation frequencies were 49.2% in MSI-High, and 4.5% in MSS tumors. A similar pattern of mutation frequencies was observed for CREBBP and EP300 in hypermutated CRC groups, resembling the mutation frequency profile of POLE and POLD1 (TMB-High: 40.7%, TMB-Low: 2.0%; MSI-High: 47.7%, MSS: 3.1%) (Figure 1).

Both CREBBP/EP300-MUT and POLE/POLD1-MUT tumors were significantly associated with right-sided colorectal cancer localization and displayed similar histopathological features, including enrichment for medullary and mucinous adenocarcinoma subtypes. Comprehensive clinical and histopathological characteristics of these molecular subgroups are summarized in Supplementary File S1 (Figures S1 and S2).

The associations between CREBBP/EP300-MUT and POLE/POLD1-MUT status and TMB-high and MSI-high phenotypes in the overall MSK-CHORD CRC cohort are likewise reported in Supplementary File S1.

### 2.2. Analysis of TMB Levels in Relation to CREBBP/EP300 and POLE/POLD1 Mutational Status in the MSK-CHORD MSS Cohort

**Figure 2:**
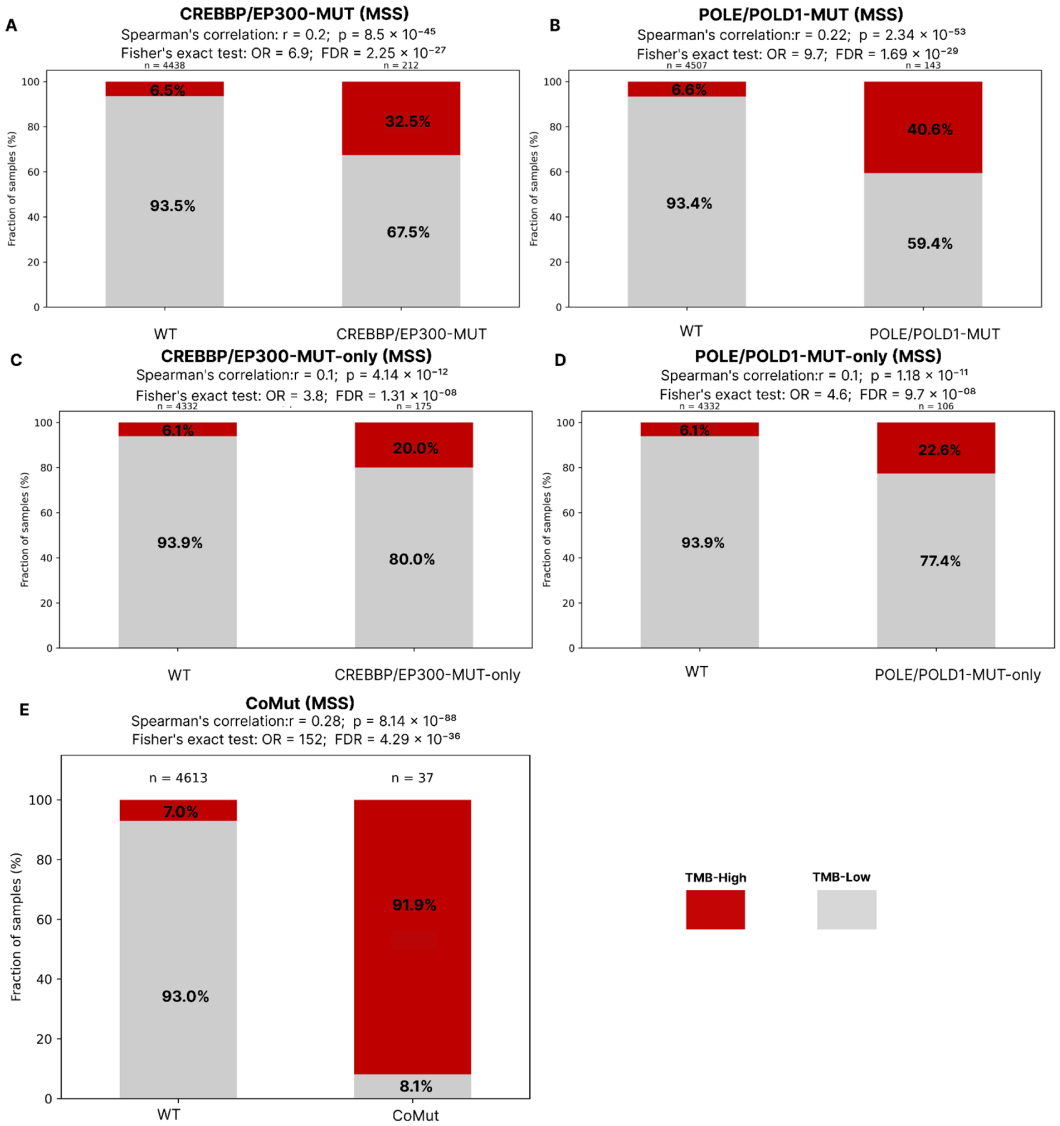
Interplay between CREBBP/EP300 and POLE/POLD1 mutations in shaping TMB in MSS MSK-CHORD cohort. (**A**) Samples stratified into two groups based solely on CREBBP and/or EP300 mutation status. (**B**) Samples stratified into two groups based solely on POLE and/or POLD1 mutation status. (**C**) Samples stratified into two groups based on CREBBP and/or EP300 mutation status, excluding those co-mutated with POLE and/or POLD1. (**D**) Samples stratified into two groups based on POLE and/or POLD1 mutation status, excluding those co-mutated with CREBBP and/or EP300. (**E**) Only the CoMut group versus all other samples. In the CoMut subgroup only three samples were TMB-low, with TMB values of 6.6, 6.9 and 9.84 Mut/Mb.

We separately assessed the role of mutational status in CREBBP and EP300, POLE and POLD1 genes within the MSS cohort. To determine the contribution of isolated and co-occurring mutations, samples were stratified into CREBBP/EP300-MUT-only, POLE/POLD1-MUT-only, and CoMut groups (Figure 2).

The CoMut subgroup showed the strongest association with TMB-high status. Specifically, 91.9% (34/37) of CoMut tumors had TMB ≥10 Mut/Mb, whereas isolated CREBBP/EP300 or POLE/POLD1 mutations retained a weaker, although statistically significant, association after exclusion of CoMut cases (Figure 2C–E).

In the MSK-CHORD CRC cohort, the mutation groups under study were extensively co-mutated with each other: 42.93% of CREBBP/EP300-mutant samples also carried mutations in POLE and/or POLD1, and conversely, 49.70% of POLE/POLD1-mutant samples harboured mutations in CREBBP and/or EP300. POLE and POLD1 ranked among the top 20 genes that significantly co-mutate with CREBBP and/or EP300 in CRC (Fisher’s exact test, POLE: OR = 12.2, FDR = 3.5 × 10^−79^; POLD1: OR = 12.6, FDR = 2.07 × 10^−71^; Supplementary Table S4). Importantly, POLE/POLD1 EDMs were markedly enriched in MSS CRC CoMut samples, being detected in 89% of cases compared with 22% of MSI-High CRC CoMut samples.

**Figure 3:**
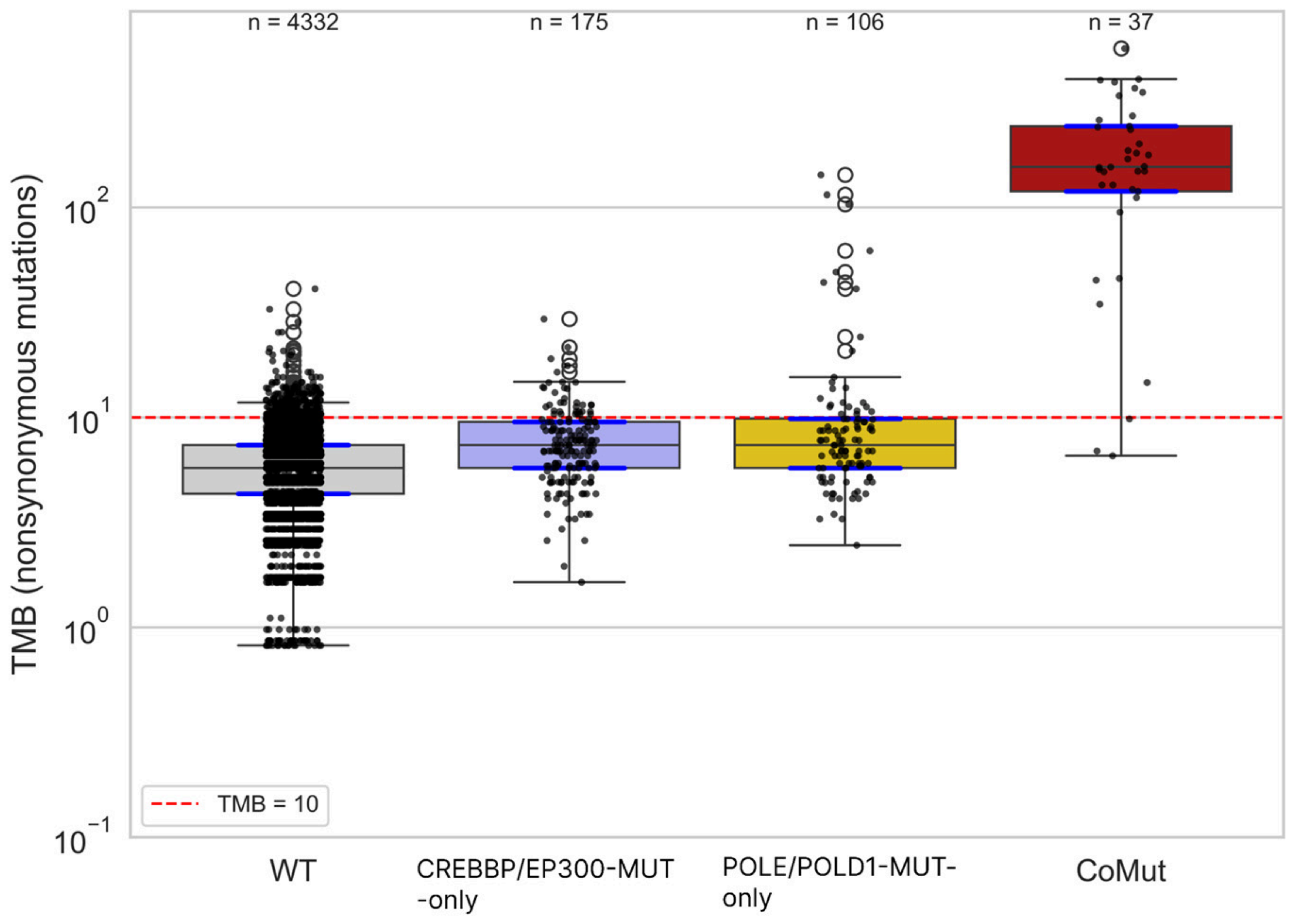
TMB distribution across mutation subgroups in the MSS subset of the MSK-CHORD cohort. The red dashed line indicates the TMB cut-off of 10 Mut/Mb.

The distribution of TMB values across mutational groups in the MSS cohort is shown in Figure 3.

This analysis revealed a highly significant overall difference in TMB across the four mutational groups (Kruskal–Wallis test: H = 201.44, *p*-value = 2.06 × 10^−43^) (Figure 3). Median TMB values increased modestly from WT tumors (median 5.74, IQR 4.32–7.38; *n* = 4332) to CREBBP/EP300-MUT-only (median 7.38, IQR 5.74–9.51; *n* = 175) and POLE/POLD1-MUT-only tumors (median 7.38, IQR 5.74–9.83; *n* = 106). Strikingly, the CoMut group showed an extreme hypermutator phenotype (median 155.87, IQR 119.33–243.84; *n* = 37), exceeding all other groups by more than an order of magnitude.

Post hoc Dunn testing confirmed that CoMut tumors had significantly higher TMB compared with all other categories (all *p* < 2.3 × 10^−7^). WT tumors consistently exhibited the lowest TMB values, with highly significant differences compared with all non-WT groups (*p* < 5.2 × 10^−14^). No significant difference was observed between CREBBP/EP300-MUT-only and POLE/POLD1-MUT-only tumors (*p* = 1.0), indicating similar TMB elevation when either CREBBP/EP300 or POLE/POLD1 is altered alone. Together, these findings establish CoMut tumors as a distinct hypermutated molecular subset, with TMB levels far exceeding those seen in tumors harboring mutations in either CREBBP/EP300 or POLE/POLD1 alone.

### 2.3. TMB and Mutational Status of CREBBP/EP300 and POLE/POLD1 in MSS TCGA COADREAD and Pan-Cancer MetTropism Cohorts

We repeated the analysis of TMB distribution in two independent cohorts. This was done to assess the reproducibility of the observed effect in both an additional CRC dataset and a pan-cancer cohort.

**Figure 4:**
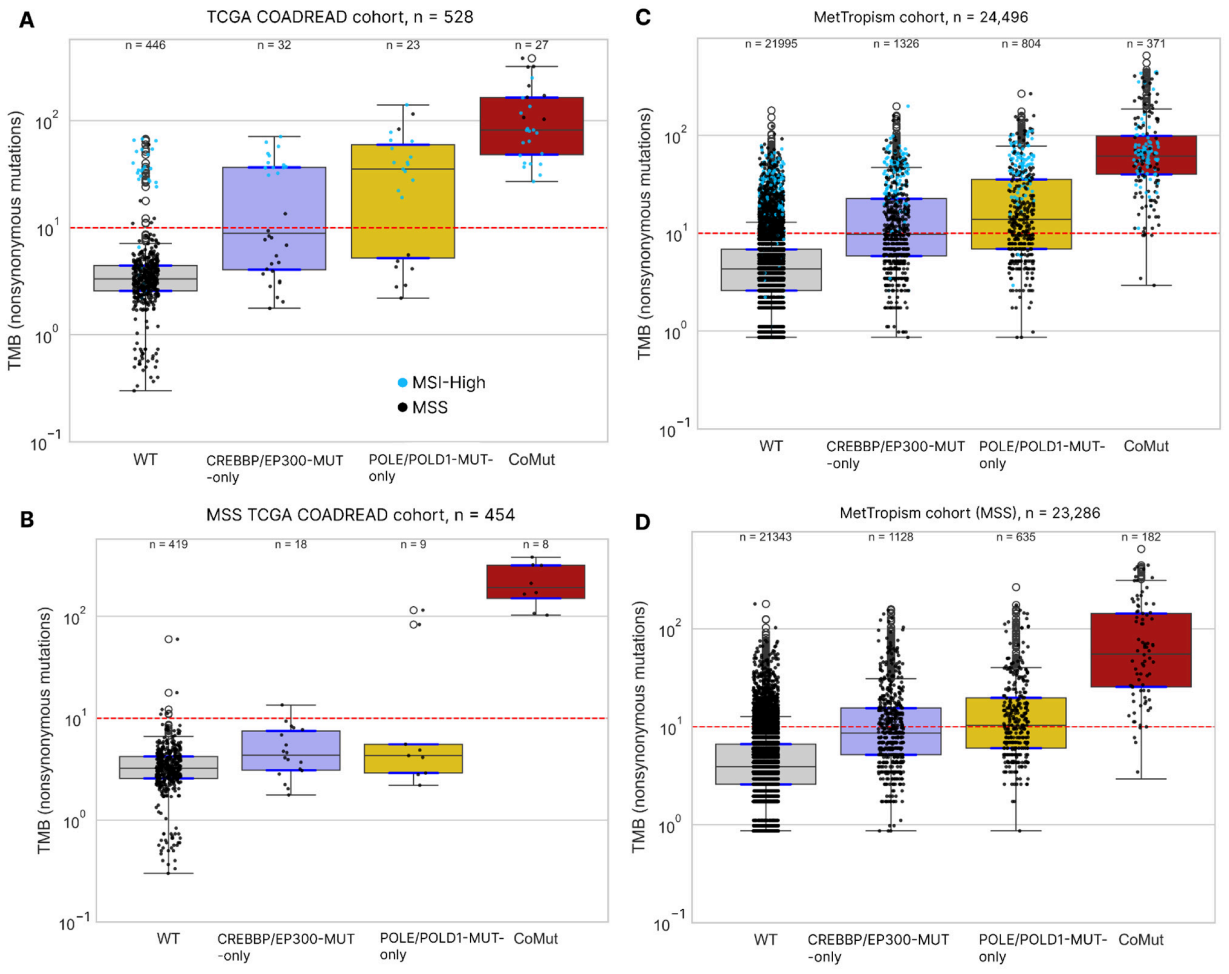
TMB distribution across mutation subgroups in the TCGA COADREAD and MetTropism cohort. (**A**) Overall TCGA COADREAD cohort (*n* = 528). (**B**) MSS subset of TCGA COADREAD cohort (*n* = 454). (**C**) Overall MetTropism cohort (*n* = 24,496). (**D**) MSS subset of MetTropism cohort (*n* = 23,286). Samples with MSI-High status are shown in blue, whereas MSS samples are shown in black. The red dashed line indicates the TMB cut-off of 10 Mut/Mb.

The distribution of TMB across mutational subgroups in the TCGA COADREAD dataset is shown below for both the full cohort (*n* = 528) and the MSS subset (*n* = 454) (Figure 4).

The plots illustrate the loss of predictive power of the isolated mutational status of the studied genes (CREBBP/EP300-MUT-only and POLE/POLD1-MUT-only) in the MSS subset of the TCGA cohort, in contrast to the CoMut group.

Given the rarity of these mutations and the limited number of samples with co-mutations, the analysis was repeated in the pan-cancer MetTropism cohort (*n* = 24,496) (Figure 4C,D). In the pan-cancer analysis, the result was reproduced: the CoMut group distinctly segregated TMB-high samples in both the overall and MSS cohorts. Similar distribution patterns were observed across individual cancer types, including CRC, bladder cancer, and other tumour types (Supplementary File S2 (Figures S3 and S4)).

Across the TCGA COADREAD and MetTropism cohorts, tumors harboring isolated CREBBP/EP300 or POLE/POLD1 mutations with extreme TMB values were predominantly observed among MSI-high cases. In contrast, the CoMut subgroup consistently exhibited exceptionally high TMB values irrespective of MSI status, including within MSS tumors (Figure 4 and Supplementary Figure S3).

### 2.4. Prognostic Effect Co-Mutated Subgroup in the MSS CRC MSK-CHORD and Pan-Cancer MetTropism Cohorts

The availability of metadata and participant numbers in the MSK-CHORD CRC and pan-cancer MetTropism cohorts enabled us to investigate the prognostic effect of the examined mutational subgroups on OS in MSS tumours. OS was analysed in the MSS CRC MSK-CHORD cohort (*n* = 4650) across the CoMut, CREBBP/EP300-MUT-only, POLE/POLD1-MUT-only and WT groups.

Median overall survival (mOS) was 47.7 months for WT, 46.9 months for CREBBP/EP300-MUT-only, and 41.2 months for POLE/POLD1-MUT-only, whereas for CoMut, the mOS was not reached (NR). The 1-year, 3-year and 5-year survival rates were 85.2%, 58.1% and 43.5% for WT; 84.6%, 58.9% and 38.7% for CREBBP/EP300-MUT-only; 88.8%, 50.4% and 43.9% for POLE/POLD1-MUT-only; and 86.1%, 79.7% and 73.6% for CoMut.

**Figure 5:**
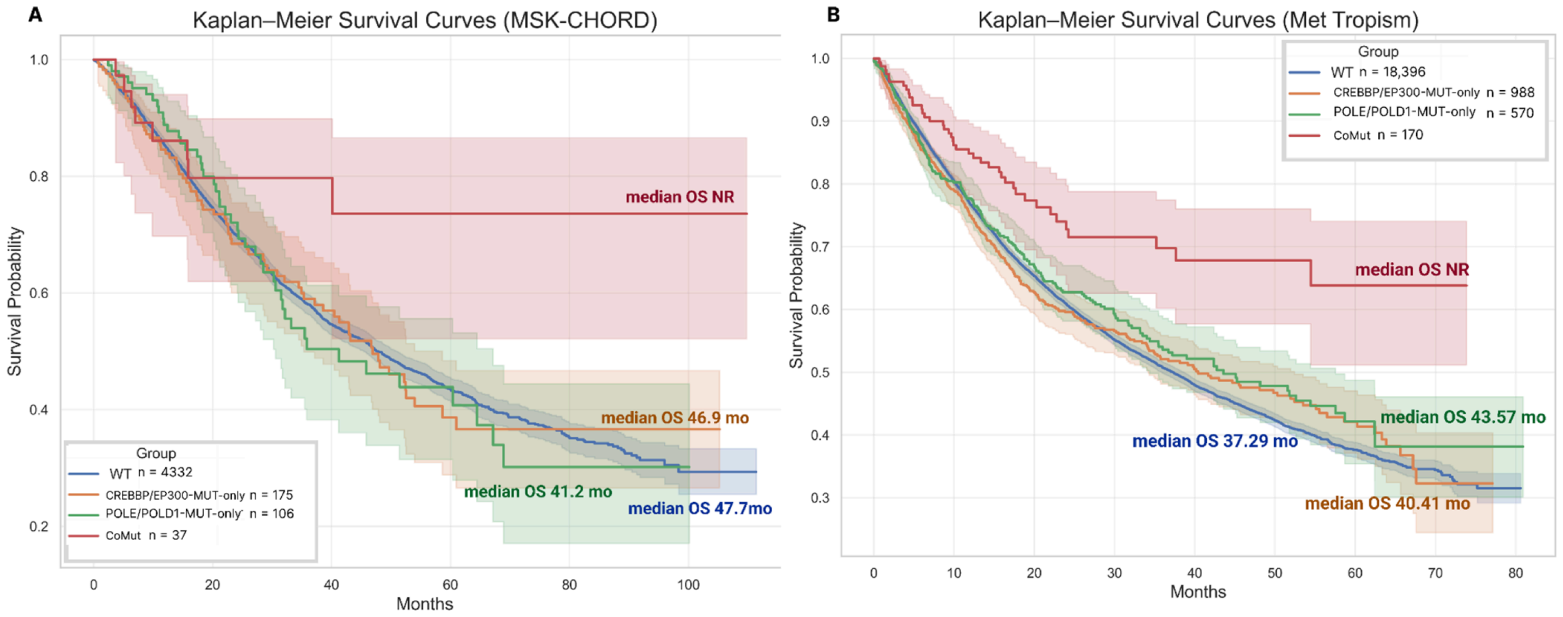
Prognostic significance of mutational status in target genes in MSS CRC (MSK-CHORD) and pan-cancer MSS (MetTropism) cohorts. (**A**) Kaplan–Meier curves for overall survival in the MSS MSK-CHORD cohort. (**B**) Kaplan–Meier curves for overall survival in the MSS MetTropism cohort. NR, median overall survival not reached within the available follow-up period, indicating that more than half of the patients were still alive at the last observation.

The overall log-rank test did not reveal statistically significant differences between the groups (χ^2^ = 4.91, *p* = 0.179); however, pairwise log-rank comparisons showed improved survival for CoMut compared with WT (*p* = 0.028) and the other subgroups. In multivariable Cox regression adjusted for age and sex, only the CoMut subgroup demonstrated a statistically significant association with improved overall survival compared to wild-type tumors (HR = 0.57, 95% CI: 0.33–0.96, *p* = 0.035), whereas neither CREBBP/EP300 nor POLE/POLD1 mutations alone showed independent prognostic value (Figure 5).

The analysis was repeated in the larger pan-cancer MetTropism cohort (Figure 5B), in which each subgroup contained a sufficient number of samples with target mutations for statistical calculations. Median OS was 37.3 months for WT, 40.4 months for CREBBP/EP300-MUT-only, and 43.6 months for POLE/POLD1-MUT-only, whereas for CoMut the median was not reached. The 1-year, 3-year and 5-year survival rates were 76.8%, 50.7% and 37.6% for WT; 75.0%, 52.1% and 42.1% for CREBBP/EP300-MUT-only; 77.7%, 53.6% and 42.2% for POLE/POLD1-MUT-only; and 84.9%, 69.8% and 63.8% for CoMut. The overall log-rank test revealed statistically significant differences between the groups (χ^2^ = 17.31, *p* = 6.09 × 10^−4^). Pairwise log-rank comparisons demonstrated improved survival for CoMut compared with WT (*p* = 6.7 × 10^−5^), CREBBP/EP300-MUT-only (*p* = 1.45 × 10^−4^) and POLE/POLD1-MUT-only (*p* = 1.70 × 10^−3^).

In the CoMut group of the MetTropism cohort, a consistently lower risk of death was observed in multivariable Cox regression adjusted for age, sex, and tumor type (HR = 0.73, 95% CI: 0.59–0.91, *p* = 0.004), in agreement with the findings from the MSK-CHORD cohort. In contrast, neither CREBBP/EP300-MUT-only nor POLE/POLD1-MUT-only tumors demonstrated independent prognostic value after adjustment (HR = 0.99 and 0.94, respectively). These results indicate that the prognostic effect is specific to the co-mutated genomic state rather than to individual gene alterations. Although tumor type accounted for a substantial proportion of survival variability, the CoMut-associated survival benefit remained robust. The relatively small size of the CoMut subgroup should still be considered when interpreting effect size estimates.

**Figure 6:**
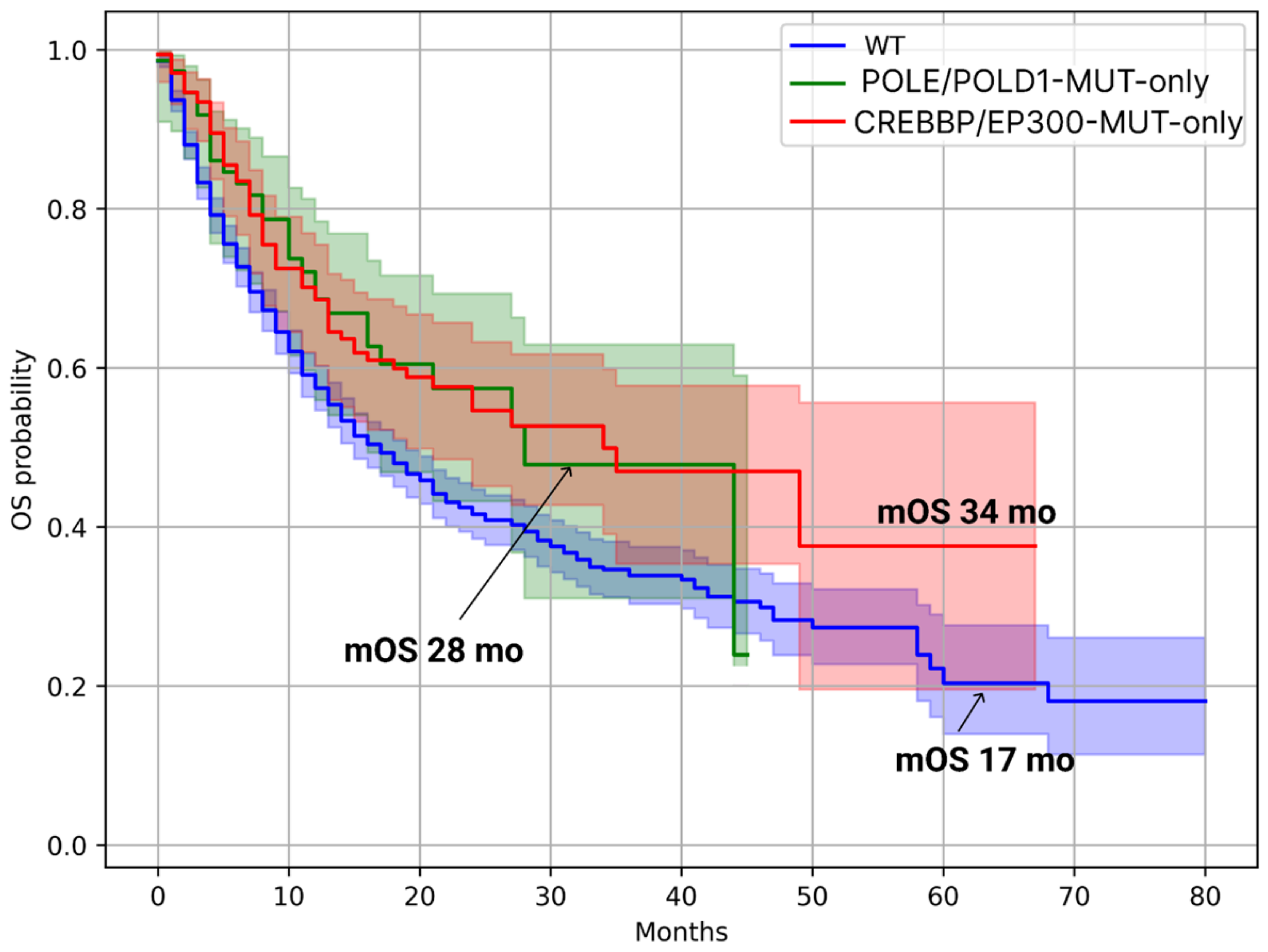
Overall survival analysis in the pan-cancer cohort of patients treated with ICIs.

We additionally analysed a pan-cancer cohort in which all patients received immunotherapy with PD-1 inhibitors with or without CTLA-4 inhibitors. Notably, no CoMut samples were present in this cohort; therefore, three groups were compared: CREBBP/EP300-MUT-only, POLE/POLD1-MUT-only and WT (Figure 6).

Overall survival analysis in the ICI-treated cohort demonstrated that both the CREBBP/EP300-MUT-only (*n* = 172) and POLE/POLD1-MUT-only (*n* = 75) groups showed survival patterns broadly comparable to each other (log-rank *p* = 0.97), without evidence of a significant difference in outcomes between the two mutational subgroups.

In comparison with the WT group, the CREBBP/EP300-MUT-only subgroup showed a significantly improved overall survival (mOS = 34 months vs. 17 months; log-rank *p* = 0.0015) and a reduced risk of death in multivariable Cox regression adjusting for age, sex, and tumor type (HR = 0.71; 95% CI: 0.56–0.88; *p* = 0.0025).

The POLE/POLD1-MUT-only subgroup also demonstrated improved overall survival compared to WT in univariable analysis (mOS = 28 months vs. 17 months; log-rank *p* = 0.033); however, this association was attenuated in the multivariable model and did not reach statistical significance after adjustment for clinical covariates (HR = 0.75; 95% CI: 0.55–1.04; *p* = 0.088).

Consistently, the CREBBP/EP300-MUT-only group exhibited the most favorable long-term outcomes, with 1-, 3-, and 5-year survival rates of 69%, 47%, and 38%, respectively, compared to WT (57%, 34%, and 20%) and POLE/POLD1-MUT-only (69%, 48%, and 24%), supporting a sustained survival advantage in CREBBP/EP300-mutant tumors under ICI therapy.

### 2.5. Differential Gene Expression Analysis and Pathway Enrichment Analysis Between Mutation Groups and WT in the TCGA Pan-Cancer Cohort

To investigate the transcriptional consequences of CREBBP/EP300 and POLE/POLD1 alterations, we analyzed RNA-seq data from the TCGA pan-cancer cohort (*n* = 7628). Compared with WT tumors, all three mutational groups (CoMut, CREBBP/EP300-MUT-only, and POLE/POLD1-MUT-only) exhibited significantly higher expression of the immune checkpoint genes PD-L1 (CD274), CTLA4, LAG3, and TIGIT, as well as the cytotoxic T-cell markers CD8A, CD8B, and IFNG (Mann–Whitney test with Bonferroni correction, all *p* < 0.001; Supplementary File S3 (Figures S5 and S6)). No significant differences in the expression of these genes were observed between the three mutational groups.

Heatmap analysis of a broader panel of immune-related genes demonstrated a consistent increase in the expression of markers associated with cytotoxic T-cell infiltration, IFN-γ signaling, immune checkpoint activation, and antigen presentation across all mutation groups (Supplementary File S3). Although the CoMut group showed relatively lower expression of several antigen presentation genes compared with the isolated mutation groups, the overall immune transcriptional profile remained highly similar across the three groups.

KEGG-based GSEA further demonstrated a shared immune-activated transcriptional phenotype across all mutational groups, with significant enrichment of pathways related to T-cell receptor signaling, natural killer cell-mediated cytotoxicity, antigen processing and presentation, interferon signaling, mismatch repair, homologous recombination, nucleotide excision repair, and Fanconi anemia (all FDR q < 0.05). Conversely, neuronal signaling pathways, including neuroactive ligand–receptor interaction, GABAergic synapse, glutamatergic synapse, calcium signaling, and cGMP-PKG signaling, were consistently depleted (all FDR q < 0.05). Direct pairwise comparisons revealed no significant differences in KEGG pathway enrichment among the three mutational groups, supporting a convergent immune-activated transcriptional phenotype. Complete differential expression and pathway enrichment results are provided in Supplementary Table S2.

## 3. Discussion

In this study, we systematically demonstrated that somatic coding mutations in the epigenetic regulator genes CREBBP and EP300 define a distinct molecular subgroup of CRC characterized by a hypermutator phenotype, immune activation, and improved survival outcomes following ICI therapy (Figure 1, Figure 2, Figure 3, Figure 4 and Figure 6; Supplementary Files S1–S3). In the MSK-CHORD cohort (Supplementary Table S1), CREBBP/EP300-MUT tumors were 6–10-fold enriched among MSI-High/TMB-High cases relative to MSS/TMB-Low tumors (Figure 1 and Figure 2), and this enrichment was accompanied by markedly higher mean and median TMB and MSIsensor values in CREBBP/EP300-MUT than in WT tumors (Supplementary File S1). A strong positive association with TMB-High and MSI-High was confirmed by Spearman’s correlation and Fisher’s exact test (Supplementary File S1). The clinicopathological features of CREBBP/EP300-MUT tumors, including a predominance of right-sided tumors and enrichment of lymphocyte-infiltrated medullary histotype, are consistent with current representations of the immunogenic CRC landscape (Supplementary File S1).

Our results show for the first time that, in both the full cohort (Figure 1, Supplementary File S1) and the MSS subset (Figure 2, Figure 3, Figure 4, Figure 5 and Figure 6, Supplementary Files S2 and S3), the CREBBP/EP300-MUT group displays clinical and molecular features that mirror those of the POLE/POLD1-MUT group. This pattern is reflected in the comparable proportions of hypermutated tumours across the mutational groups, the similarly strong associations with TMB-high/MSI-high status, and the persistence of these similarities across mOS analyses in both the prognostic (Figure 5) and immunotherapy-treated settings (Figure 6). However, in the ICI-treated cohort, multivariable Cox regression adjusted for age, sex, and tumor type showed that only the CREBBP/EP300-MUT-only subgroup retained an independent association with a reduced risk of death (HR = 0.71; 95% CI: 0.56–0.88; *p* = 0.0025), whereas the association for the POLE/POLD1-MUT-only subgroup was attenuated and no longer statistically significant (HR = 0.75; 95% CI: 0.55–1.04; *p* = 0.088). Thus, despite comparable unadjusted survival benefits, isolated defects in histone acetyltransferases CREBBP/EP300 may possess a more pronounced independent predictive value for immunotherapy than polymerase mutations. Subgroup analysis within individual tumor types was previously conducted by us, demonstrating a significant improvement in mOS for CREBBP/EP300-MUT-only in CRC [30]. Intriguingly, this same patient cohort was previously used to validate POLE/POLD1 mutations as biomarkers of immunotherapy response [6], providing a rationale for subsequent clinical trials.

Previous studies have demonstrated that POLE/POLD1-mutated CRCs exhibit high CD8+ T-cell infiltration, PD-L1 expression, and other features characteristic of an immunologically “hot” tumor microenvironment (TME) [31,32,33]. In our work, in the TCGA pan-cancer cohort, sample groups without mutual co-mutations of the studied epigenetic modifiers and polymerases (i.e., CREBBP/EP300-MUT-only and POLE/POLD1-MUT-only) exhibit similar patterns of a hot TME and overexpression of key immune checkpoints in contrast to WT (Supplementary File S3, Supplementary Table S2).

We observed a strong mutual co-mutation between CREBBP/EP300-MUT and POLE/POLD1-MUT (Figure 1 and Figure 2, Supplementary Table S4)—more than 40% of each group harbours a co-mutation. To evaluate the independent contribution of mutations in epigenetic modifiers and the studied polymerases, we isolated the CoMut group. This led to a reassessment of the role of POLE and POLD1 mutations in MSS CRC. Specifically, CoMut exhibited a pronounced synergistic effect, enabling robust identification of TMB-High tumours in MSS CRC: 91% TMB-High compared with 20% and 22.6% in the respective isolated mutation groups (CREBBP/EP300-MUT-only and POLE/POLD1-MUT-only). Exclusion of CoMut samples from the CREBBP/EP300-MUT and POLE/POLD1-MUT groups led to a marked parallel decrease in both the proportion of hypermutated tumours and the strength of the association with TMB-High within the MSS subsets (Figure 2).

CoMut was characterised by ultra-high TMB values across all studied cohorts (Figure 2, Figure 3 and Figure 4) and exhibited the greatest predictive strength in regression models in both the CRC cohort and the pan-cancer cohort (Supplementary File S2). In the MSS subsets of the CRC and pan-cancer cohorts, neither POLE/POLD1-MUT-only nor CREBBP/EP300-MUT-only status was independently predictive of hypermutated tumours in regression models (Supplementary File S2).

It was not possible to assess the efficacy of immunotherapy in the CRC and pan-cancer cohorts within the CoMut group; however, in the MSS MSK-CHORD and MetTropism cohorts, we demonstrate an overall positive prognostic effect on mOS (Figure 5). CoMut, as well as CREBBP/EP300-MUT-only and POLE/POLD1-MUT-only, demonstrates high expression of immune checkpoints (PD-L1, CTLA4, LAG3, TIGIT), CD8A/CD8B, and IFNG; however, decreased expression of a gene cluster responsible for antigen presentation is observed, which is expected for the group with ultra-hypermutated tumours (Supplementary File S3).

In routine clinical practice, early molecular profiling of colorectal cancer is commonly performed using targeted NGS panels covering clinically relevant genes, such as KRAS, NRAS, and BRAF, which increasingly include POLE and POLD1. Our findings suggest that extending such panels to include CREBBP and EP300 may provide incremental value for identifying the rare CoMut molecular phenotype. If validated in prospective clinical studies, this approach could facilitate earlier recognition of MSS tumors with an exceptionally high mutational burden, without the need to rely on comprehensive genomic profiling as the initial molecular test.

Collectively, these findings position CREBBP and EP300 mutations as clinically relevant biomarkers comparable to POLE/POLD1 for identifying hypermutated CRC. The CoMut subgroup defines an ultra-hypermutated MSS population that may serve as a surrogate marker of TMB-high status, potentially reducing reliance on direct TMB testing. The replication of these findings across independent cohorts, including pan-cancer ICI-treated populations, highlights a functional interplay between epigenetic dysregulation and DNA repair deficiency in shaping tumor immunogenicity and therapeutic response.

## 4. Materials and Methods

### 4.1. Study Cohorts and Subgroups

The primary discovery cohort consisted of the CRC data MSK-CHORD cohort (*n* = 5493). Independent validation was performed using the TCGA COADREAD cohort (*n* = 528) and the pan-cancer MetTropism cohort (*n* = 24,496). Somatic mutation profiles, MSIsensor scores, TMB estimates, and available clinical metadata were obtained from cBioPortal (https://www.cbioportal.org/, accessed on 10 October 2025). Only samples with both available somatic mutation profiles and corresponding clinical metadata in the original cBioPortal datasets were included in the analysis.

Standard thresholds were applied to define MSI-high (MSIsensor score ≥ 3.5 [34,35]) and TMB-high (≥10 Mut/Mb [36]). Additional TMB cutoffs (≥20 and ≥50 Mut/Mb) were used in selected analyses. The ≥20 Mut/Mb threshold has been proposed in several studies as an alternative cutoff for identifying colorectal cancers with a higher mutational burden, whereas the ≥50 Mut/Mb threshold has been used to define ultra-hypermutated tumors in previous landmark studies and clinical guidelines [12,37,38].

Samples were stratified based on the presence of coding somatic mutations (missense, nonsense, frameshift, and splice-site mutations) in CREBBP and EP300. No additional filtering according to predicted functional consequence or pathogenicity was applied, as the aim of the study was to evaluate mutation status as a clinically applicable biomarker. Tumors were classified into four mutually exclusive groups: CREBBP-MUT (mutations in CREBBP only), EP300-MUT (mutations in EP300 only), CREBBP/EP300-MUT (mutations in both genes), and CREBBP/EP300-WT (no mutations in either gene). For selected analyses, tumors harboring mutations in CREBBP and/or EP300 were collectively referred to as CREBBP/EP300-MUT.

For additional stratification, tumors were further classified based on the presence of mutations in POLE and/or POLD1 (POLE/POLD1-MUT). Polymerase-mutated tumors were subsequently subdivided according to mutation localization into those affecting the exonuclease domain (EDM-POLE/POLD1-MUT) and those located outside this domain (non-EDM-POLE/POLD1-MUT).

The CoMut subgroup was defined as tumors harboring concurrent mutations in CREBBP and/or EP300 together with POLE and/or POLD1. Sample groups with mutations, excluding those in the CoMut subgroup, were designated as CREBBP/EP300-MUT-only and POLE/POLD1-MUT-only, respectively.

The comprehensive clinical metadata and mutational calls for all samples analyzed in this study are consolidated in Supplementary Table S1.

### 4.2. Statistical Analysis

Statistical analyses were performed in Python (v3.13.5). Associations between categorical variables were assessed using Fisher’s exact test, with odds ratios (ORs) and 95% confidence intervals (CIs) reported where applicable. Differences between two groups were evaluated using the Mann–Whitney U test or Welch’s *t*-test, whereas comparisons across multiple groups were performed using the Kruskal–Wallis test followed by Dunn’s post hoc test. Correlations were assessed using Spearman’s rank correlation coefficient. Bonferroni correction was applied for multiple testing. All statistical tests were two-sided, and *p* < 0.05 was considered statistically significant unless otherwise specified.

To evaluate the independent contribution of genomic alterations to tumor mutational burden (TMB), multiple linear regression models were fitted using log2-transformed TMB values (log2[TMB + 1]) as the dependent variable. Mutation status was modeled as four mutually exclusive groups: CREBBP/EP300-MUT-only, POLE/POLD1-MUT-only, CoMut, and WT (reference group). Models were adjusted for available clinical and molecular covariates, including age, sex, MSI status, and KRAS, NRAS, and BRAF mutation status where applicable.

Model interpretation was complemented by SHapley Additive exPlanations (SHAP) analysis. Mean absolute SHAP values were used to estimate the relative contribution of individual predictors to TMB variation and to assess the consistency of feature importance with regression-derived effect estimates.

### 4.3. Survival Analysis

Overall survival (OS) was estimated using the Kaplan–Meier method, and differences between mutation-defined subgroups were evaluated with the log-rank test. Multivariable Cox proportional hazard regression was performed to evaluate the association between mutation status and OS, adjusting for age, sex, and tumor type. Median survival times, one-, three-, and five-year survival rates, hazard ratios (HRs), and 95% CIs were reported. All tests were two-sided, with *p* < 0.05 considered statistically significant.

The prognostic significance of the mutation-defined groups (CREBBP/EP300-MUT-only, POLE/POLD1-MUT-only, and CoMut) was evaluated in the MSS cohorts of CRC MSK-CHORD and in the pan-cancer MetTropism cohort.

The OS analysis and the prognostic significance of CREBBP/EP300-MUT-only and POLE/POLD1-MUT-only statuses were performed in an independent clinical pan-cancer cohort treated with immunotherapy with PD-1/PD-L1 and/or CTLA-4 inhibitors (*n* = 1610) [39]. No CoMut cases were available in the analytical dataset used for survival analysis. Data for the analysis were obtained from cBioPortal (https://www.cbioportal.org/, accessed on 21 October 2025).

### 4.4. Analysis of Differential Gene Expression and Pathway Enrichment in the Pan-Cancer TCGA Cohort

To assess the transcriptional impact of CREBBP/EP300 and POLE/POLD1 mutations at a pan-cancer level, we analyzed combined somatic mutation and RNA-seq expression profiles from The Cancer Genome Atlas (TCGA) pan-cancer cohort (*n* = 7628). Expression data (log2[TPM + 0.001]) and mutation calls were obtained from the UCSC Xena browser (https://xenabrowser.net; accessed 5 January 2026). Samples lacking either expression or mutation data were excluded. Samples were stratified into four groups based on their mutational status: (1) CoMut (*n* = 94); (2) CREBBP/EP300-MUT-only (*n* = 487); (3) POLE/POLD1-MUT-only (*n* = 209); and (4) Wild Type (WT, *n* = 6838), which served as a common reference.

We compared the expression levels of pre-selected key genes of TIME associated with immunotherapy efficacy (CD3D, CD3E, CD8A, CD8B, MS4A1, CD79A, NKG7, KLRD1, CD68, CSF1R, CD163, CD274, PDCD1, CTLA4, LAG3, TIGIT, HAVCR2, IFNG, CXCL9, CXCL10, CXCL11, STAT, GZMB, HLA-A, HLA-B, HLA-C, B2M, TAP1, TAP2) between each mutational group and the WT group. Gene expression levels were compared using the non-parametric Mann–Whitney U test with Bonferroni adjustment.

To investigate broader transcriptional programs, we performed a Kyoto Encyclopedia of Genes and Genomes (KEGG) pathway-based Gene Set Enrichment Analysis (GSEA) [40,41]. The analysis was conducted by contrasting the gene expression profile of each mutational group (CoMut, CREBBP/EP300-MUT-only, POLE/POLD1-MUT-only) against the WT group. Enriched and depleted pathways were identified based on a false discovery rate (FDR) q-value < 0.05. To assess the similarity of pathway alterations between the three mutational groups, their respective GSEA enrichment profiles were systematically compared. Direct pairwise GSEA comparisons between the mutational groups were also performed to identify potential significant differences.

The full results of the TIME gene expression analysis and GSEA are presented in Supplementary Table S2.

## 5. Conclusions

Somatic mutations in CREBBP and EP300 exhibit clinicogenomic features comparable to POLE/POLD1-mutant tumors and are associated with improved survival after immune checkpoint inhibitor therapy, supporting their prospective evaluation as predictive biomarkers for immunotherapy stratification. Co-occurring CREBBP/EP300 and POLE/POLD1 mutations (CoMut) define a microsatellite-stable CRC subset with ultra-high tumor mutational burden and independently favorable prognosis, suggesting potential utility as a surrogate marker of TMB-high status pending rigorous prospective validation. These findings encourage the evaluation of both isolated CREBBP/EP300 alterations and CoMut status in biomarker-guided clinical trials.

## Data Availability

The datasets analysed during the current study are publicly available. Somatic mutation profiles, TMB estimates, MSIsensor scores and clinical annotations for the MSK-CHORD, TCGA COADREAD, MetTropism and Samstein cohorts were obtained from cBioPortal (https://www.cbioportal.org (accessed on 10 October 2025)). Pan-cancer RNA-seq and mutation data were downloaded from the UCSC Xena browser (https://xenabrowser.net/datapages/ (accessed on 15 January 2026)). All processed data generated in this study are provided in the Supplementary Information (Tables S1–S6, Files S1–S3).

## Supplementary Materials

The following supporting information can be downloaded at: https://www.mdpi.com/article/10.3390/ijms27157027/s1.

## Abbreviations

The following abbreviations are used in this manuscript:

CBP	CREB-binding protein (encoded by CREBBP)
CI	Confidence interval
CoMut	Concurrent mutations in CREBBP and/or EP300 together with POLE and/or POLD1
CRC	Colorectal cancer
CTLA-4	Cytotoxic T-lymphocyte-associated protein 4
EDM	Exonuclease domain mutations
EP300	E1A binding protein p300 gene
FDR	False discovery rate
GSEA	Gene Set Enrichment Analysis
HAT	Histone acetyltransferase
HR	Hazard ratio
ICIs	Immune checkpoint inhibitors
IHC	Immunohistochemistry
IQR	Interquartile range
KAT	Lysine acetyltransferase
KEGG	Kyoto Encyclopedia of Genes and Genomes
mOS	Median overall survival
MSI	Microsatellite instability
MSI-H	Microsatellite instability-high
MSI-L	Microsatellite instability-low
MSS	Microsatellite stable
MSK-CHORD	Memorial Sloan Kettering Cancer Center—Clinical Health Outcomes Research & Development cohort
Mut/Mb	Mutations per megabase
non-EDM	Non-exonuclease domain mutations
NR	Not reached
OR	Odds ratio
OS	Overall survival
PCR	Polymerase chain reaction
PD-1	Programmed cell death protein 1
PD-L1	Programmed death-ligand 1
POLD1	DNA polymerase delta 1 gene
POLE	DNA polymerase epsilon gene
SHAP	SHapley Additive exPlanations
TCGA	The Cancer Genome Atlas
TIGIT	T-cell immunoreceptor with Ig and ITIM domains
TIME	Tumor immune microenvironment
TMB	Tumor mutational burden
TPM	Transcripts per million
WT	Wild type
