# Supplementary material for: Co-Mutation of CREBBP/EP300 and POLE/POLD1 Identifies a TMB-High Subset of MSS CRC
Source: Int J Mol Sci. 2026 Aug 5;27(15):7027. doi: 10.3390/ijms27157027 (PMC13467031; doi:10.3390/ijms27157027)
Supplement: Supplementary file 1 [file ijms-27-07027-s001.zip › Supplementary File S1.pdf]

Supplementary File S1.

Clinicopathological features of CREBBP/EP300 and POLE/POLD1 mutant tumours

Both CREBBP/EP300-MUT and POLE/POLD1-MUT tumors were significantly associated with right-sided cancer localization. Among CREBBP/EP300-MUT 56.4% were located in the right side of the colon, whereas left-sided tumors predominated in the WT group (72.9%) ($\chi^2 = 205.64$, df = 1, $p < 0.0001$; OR = 3.48, 95% CI 2.92–4.16; Fisher exact test, $p < 0.0001$). Among POLE/POLD1-MUT 61.7% were right-sided compared with 27.0% in WT tumors ($\chi^2 = 252.06$, df = 1, p-value < 0.0001; OR = 4.36, 95% CI 3.60–5.29; Fisher exact test, $p < 0.0001$).

The significance and interpretation of mutations outside the EDM of POLE and POLD1 are currently widely debated. In our analysis, both EDM and non-EDM POLE/POLD1-mutated tumors showed a higher likelihood of right-sided localization compared with WT tumors (EDM: OR = 3.71, 95% CI 2.60–5.29; non-EDM: OR = 4.63, 95% CI 3.70–5.79; Fisher's exact test, $p < 0.0001$ for both). No statistically significant difference was observed between EDM and non-EDM mutations for this parameter (OR = 0.80, 95% CI 0.53–1.21; Fisher's exact test, $p = 0.29$). These findings suggest that right-sided localization is a common feature of POLE/POLD1-mutated tumors regardless of mutation domain, whereas WT tumors are predominantly left-sided.

We next examined the available metadata on histological tumor types in the CRC MSK-CHORD cohort and assessed their association with mutational status. The vast majority of cases in the overall cohort were classified as adenocarcinoma, NOS. A small number of samples were represented by medullary carcinoma (n = 21) and mucinous adenocarcinoma (n = 111). Notably, medullary carcinoma showed a high prevalence of tumors harboring mutations in CREBBP and/or EP300 (76.2%) as well as in POLE and/or POLD1 (61.9%). Moreover, co-mutant tumors involving both gene groups accounted for 57% of cases within this histological subtype (Supplementary Figure S1).

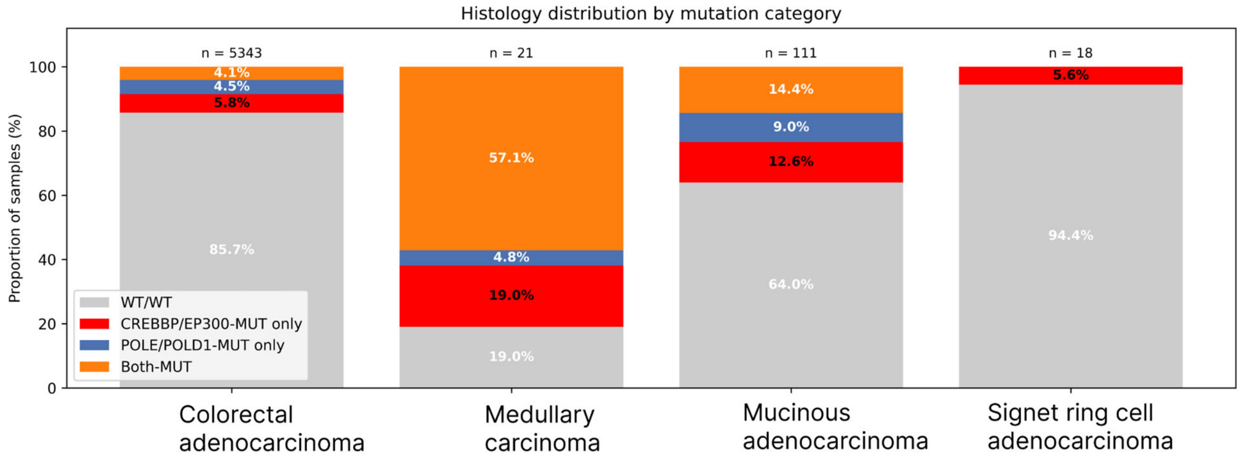

Supplementary Figure S1. Histological subtypes of colorectal cancer and mutational status of CREBBP, EP300, POLE, and POLD1. Histological distribution of combined CREBBP/EP300 and POLE/POLD1 mutation categories in colorectal cancer.

Association of CREBBP/EP300 and POLE/POLD1 mutations with TMB-high and MSI-high status in the MSK-CHORD cohort

We identified statistically significant differences in mean TMB values (60.23 vs 8.74, p-value = 2.16×10^{-55}) and MSIsensor scores (17.8 vs 0.44, p-value = 2.12×10^{-84}) between tumors harboring mutations in CREBBP and/or EP300 and WT (Welch's t-test) (Table S3-1).

Table S3. Descriptive statistics of TMB and MSI scores in CREBBP/EP300-MUT group vs WT.

Group	Mean	Median	Min	Max	Q1	Q3	IQR	N
**TMB scores**								
**CREBBP/EP300-MUT**	60.23	50.15	1.64	570.96	9.84	74.36	64.52	573
**WT**	8.74	5.87	0.82	142.89	4.32	7.78	3.46	4920
**MSI scores**								
**CREBBP/EP300-MUT**	18.16	17.98	0.00	53.53	0.73	32.54	31.80	571
**WT**	2.17	0.44	0.00	52.85	0.13	1.18	1.05	4882

A similar trend was observed when comparing TMB levels (59.07 vs 6.05, p-value = 1.37×10^{-61}) and MSIsensor scores (21.0 vs 2.14, p-value = 4.42×10^{-91}) between the POLE/POLD1-mutant and wild-type groups (Welch's t-test) (Table S3-2).

Table S5. Descriptive statistics of TMB values in POLE/POLD1 groups.

Group	Mean	Median	Min	Max	Q1	Q3	IQR	N
**TMB scores**								

**POLE/POLD1-MUT**	70.74	59.07	2.46	570.96	32.81	82.15	49.33	495
**WT**	8.50	6.05	0.82	198.68	4.32	7.78	3.46	4998
**MSI scores**								
**POLE/POLD1-MUT**	21.05	21.87	0.00	53.53	1.66	35.80	34.13	492
**WT**	2.14	0.44	0.00	51.36	0.13	1.18	1.05	4961

We further confirmed that CREBBP/EP300-MUT is significantly correlated with TMB-high status (Spearman's correlation: r = 0.50; Fisher's exact test: OR = 22.36). A similarly strong association was observed with MSI status assessed by MSIsensor (Spearman's correlation: r = 0.5; Fisher's exact test: OR = 20.60). A comparable pattern was observed for the POLE/POLD1-MUT group with respect to TMB-high status (Spearman's correlation: r = 0.52; Fisher's exact test: OR = 34.22 and MSIsensor-derived MSI status (Spearman's correlation: r = 0.52; Fisher's exact test: OR = 28.70). Detailed statistical results and figures are available in Supplementary Table S3-3.

Table S6. Summary of Spearman Correlation and Fisher's Exact Test in Mutation in general CRC MSK-CHORD cohort.

**Cohort**	**Factor**	**Spearman's rho**	**Spearman's adj. Bonf**	**Fisher OR**	**CI low**	**CI high**	**Fisher p adj. Bonf**
**MSK-CHORD**							
CREBBP/EP300-MUT	TMB-High	0.50	0.00E+00	22.36	18.17	27.52	4.91e-223
CREBBP/EP300-MUT	MSI-High	0.50	0.00E+00	20.60	16.86	25.16	1.469e-199
POLE/POLD1-MUT	TMB-High	0.52	0.00E+00	34.22	26.76	43.77	2.103e-240
POLE/POLD1-MUT	MSI-High	0.53	0.00E+00	28.70	23.04	35.76	1.269e-218
CREBBP/EP300-MUT-only	TMB-High	0.37	5.20e-156	13.56	10.65	17.26	1.345e-92
CREBBP/EP300-MUT-only	MSI-High	0.39	5.774e-175	16.63	12.89	21.46	7.3571e-90
POLE/POLD1-MUT-only	TMB-High	0.39	7.298e-177	20.07	15.15	26.59	1.258e-97
POLE/POLD1-MUT-only	MSI-High	0.43	3.504e-216	24.99	18.81	33.19	3.389e-99
CoMut	TMB-High	0.45	1.78e-273	478.96	152.994	1499.42	1.40e-184
CoMut	MSI-High	0.45	1.50e-275	49.20	34.412	70.355	8.17e-151
**MSS MSK-CHORD**							

CREBBP/EP300-MUT	TMB-High	0.20	8.49e-45	6.939	5.094	9.452	2.25e-27
POLE/POLD1-MUT	TMB-High	0.22	2.34e-53	9.697	6.838	13.752	1.69e-29
CREBBP/EP300-MUT-only	TMB-High	0.106	4.14e-12	3.792	2.567	5.602	1.31e-08
POLE/POLD1-MUT-only	TMB-High	0.104	1.18e-11	4.569	2.874	7.264	9.70e-08
CoMut	TMB-High	0.283	8.14e-88	152.893	46.776	499.745	4.29e-36

Importantly, after excluding co-mutated samples (CREBBP/EP300 + POLE/POLD1; CoMut group), the strength of the association decreased in parallel for both the CREBBP/EP300-MUT-only and POLE/POLD1-MUT-only groups. However, a strong and statistically significant association with the TMB-high phenotype was retained. The CoMut group samples (n = 246) showed exceptional concordance with the TMB-high status: 98.8% of samples had TMB > 10 Mut/Mb (Supplementary Figure S2).

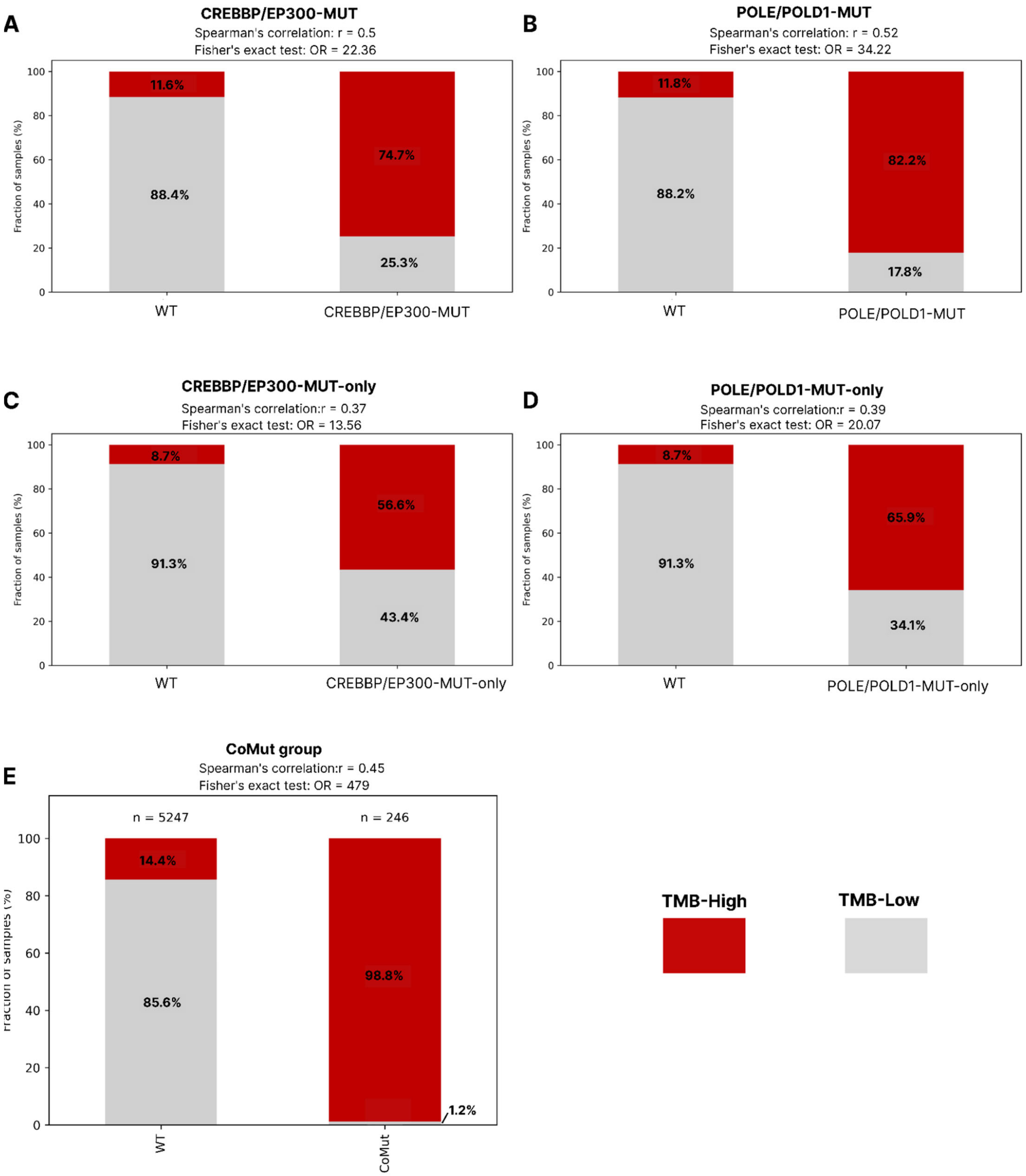

Supplementary Figure S2. Interplay between CREBBP/EP300 and POLE/POLD1 mutations in shaping TMB in CRC. **A.** Samples stratified into two groups based solely on CREBBP and/or EP300 mutation status. **B.** Samples stratified into two groups based solely on POLE and/or POLD1 mutation status. **C.** Samples stratified into two groups based on CREBBP and/or EP300 mutation status, excluding those co-mutated with POLE and/or POLD1. **D.** Samples stratified into two groups based on POLE and/or POLD1 mutation status,

excluding those co-mutated with CREBBP and/or EP300. **E.** Only the CoMut group (CREBBP\EP300 + POLE\POLD1) versus all other samples.
