# Supplementary material for: Co-Mutation of CREBBP/EP300 and POLE/POLD1 Identifies a TMB-High Subset of MSS CRC
Source: Int J Mol Sci. 2026 Aug 5;27(15):7027. doi: 10.3390/ijms27157027 (PMC13467031; doi:10.3390/ijms27157027)
Supplement: Supplementary file 1 [file ijms-27-07027-s001.zip › Supplementary File S2.pdf]

Supplementary File S2

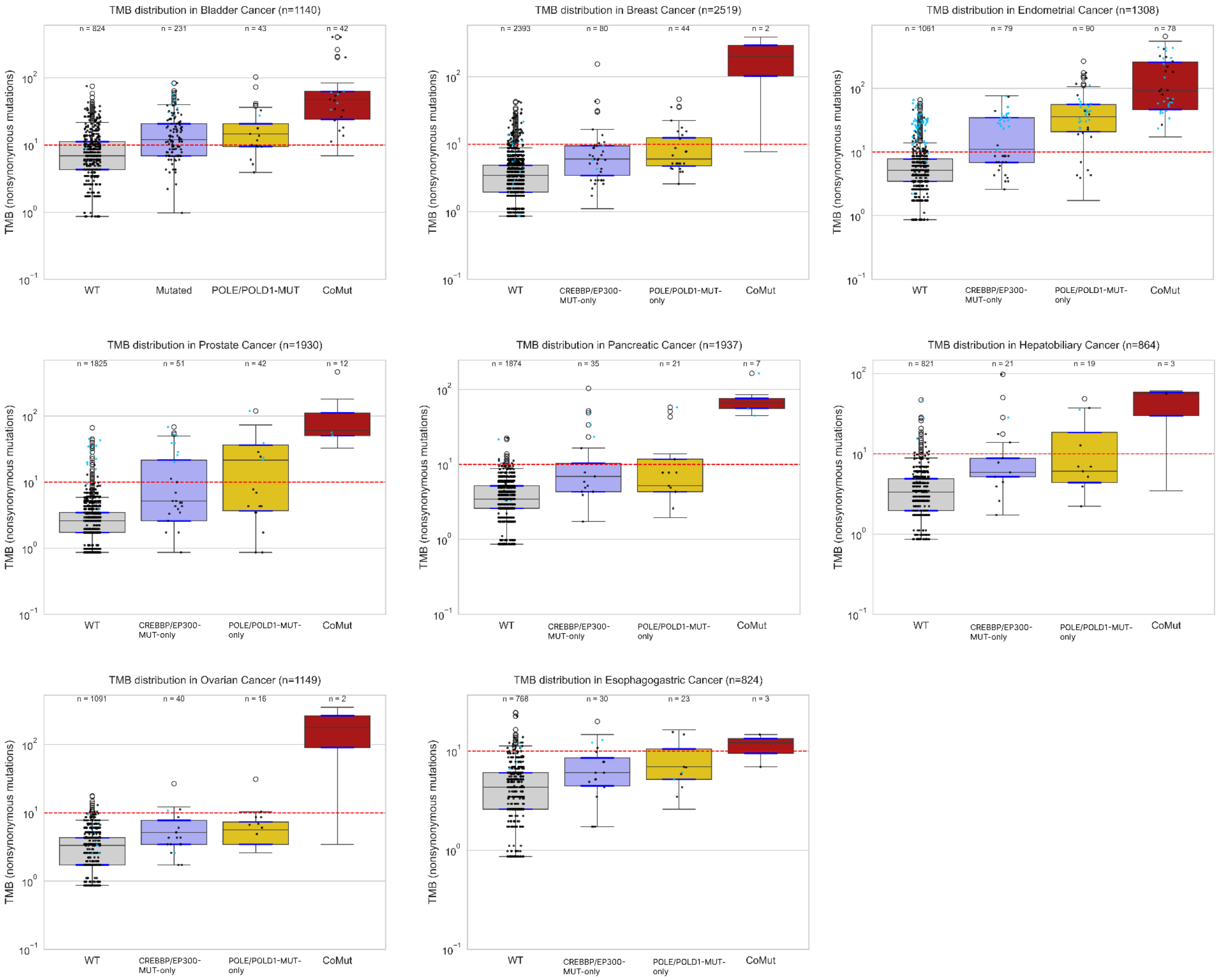

Supplementary Figure S3. TMB distribution across mutation subgroups within cancer type-specific cohorts of the MetTropism study.

Multiple linear regression in MSS cohorts

We performed multiple linear regression to assess the independent predictive capacity of mutational status in CREBBP, EP300, POLE, and POLD1. The dependent variable was the log-transformed TMB value ($\log_2$[TMB] + 1). The model included the following predictors:

CREBBP/EP300-MUT-only, POLE/POLD1-MUT-only, CoMut, WT (wild-type for all four genes), as well as sex, age, and the presence of coding mutations in KRAS, NRAS, and BRAF.

In the MSS cohort, the strongest independent molecular predictor was the CoMut group. This variable exhibited an exceptionally pronounced effect ($\beta = 3.63$, $p < 10^{-16}$; $\exp(\beta) = 37.7$), corresponding to an almost 40-fold increase in TMB compared to MSS tumours lacking these alterations. POLE/POLD1-MUT-only did not demonstrate an independent effect ($\beta = -0.04$, $p = 0.39$), indicating that an isolated proofreading repair defect in the MSS context is insufficient to drive a significant increase in mutational burden in the absence of concomitant epigenetic regulation disruptions or other mechanisms of genomic instability. The contribution of the mutation groups was confirmed by SHAP analysis (Supplementary Figure S4).

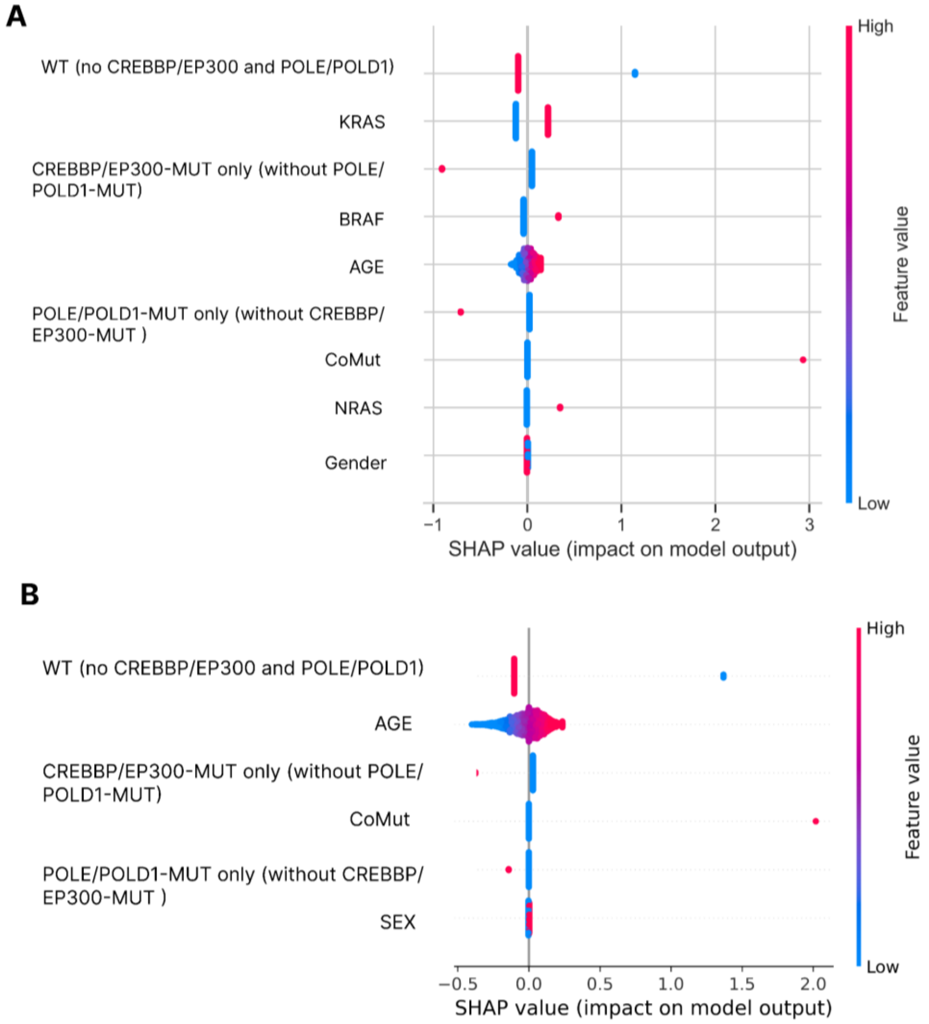

Supplementary Figure S4. SHAP analysis of clinical and genomic determinants of TMB-High status in MSS tumors. **A.** SHAP summary plot of factors associated with tumor mutational burden in MSS colorectal tumors from the MSK-CHORD cohort. **B.** SHAP summary plot showing the contribution of clinical and mutational features to TMB in MSS-only tumors from the MetTropism cohort. SHAP summary plot showing the contribution of clinical and

mutational features to predicted $\log_2$-transformed TMB in MSS colorectal tumors. Each point represents an individual tumor. The x-axis indicates SHAP values, reflecting the direction and magnitude of each feature's contribution to the model prediction, while features are ranked on the y-axis by mean absolute SHAP importance. Color denotes feature values (red = high, blue = low for continuous variables; red = 1, blue = 0 for binary variables). Binary mutational groups (CREBBP/EP300-mutant only, POLE/POLD1-mutant only, co-mutant, and wild-type) appear as vertically clustered points due to their dichotomous nature.

Given the small size of the CoMut subgroup in MSK-CHORD and TCGA COADREAD, we additionally validated our findings in the MSS group of the pan-cancer MetTropism cohort. The analysis again showed that the CoMut group remained the strongest independent molecular predictor of TMB ($\beta = 2.67$, $p < 10\text{-}16$; $\exp(\beta) = 14.5$), corresponding to a substantial increase in TMB compared to WT tumours. SHAP analysis confirmed the regression results (Supplementary Figure S4B).
