# Supplementary material for: Co-Mutation of CREBBP/EP300 and POLE/POLD1 Identifies a TMB-High Subset of MSS CRC
Source: Int J Mol Sci. 2026 Aug 5;27(15):7027. doi: 10.3390/ijms27157027 (PMC13467031; doi:10.3390/ijms27157027)
Supplement: Supplementary file 1 [file ijms-27-07027-s001.zip › Supplementary File S3.pdf]

Supplementary File S3

RNA-seq data from the TCGA pan-cancer cohort (n = 7,628) were used for differential expression and pathway enrichment analyses across WT, CREBBP/EP300-MUT-only, POLE/POLD1-MUT-only, and CoMut groups.

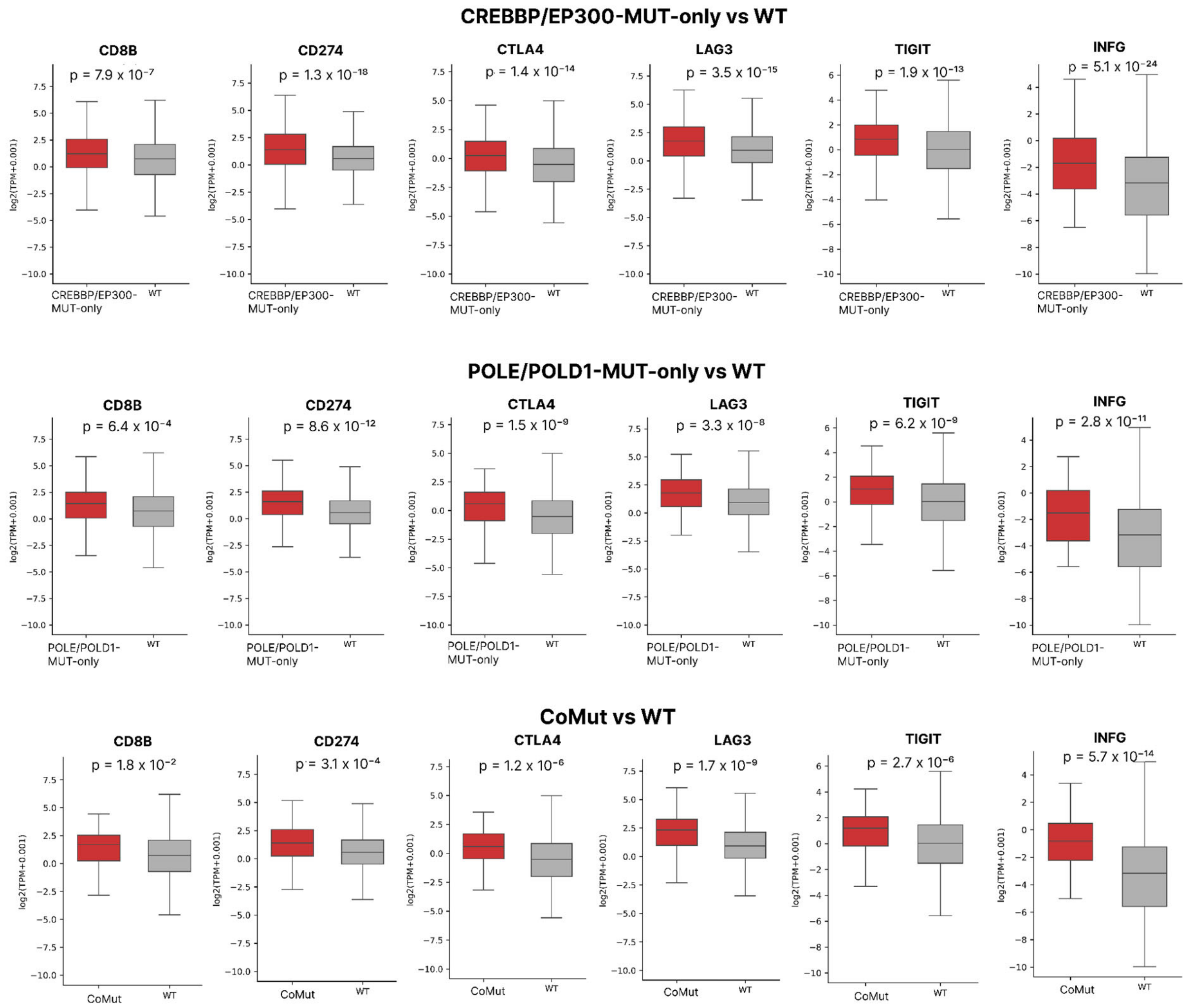

Supplementary Figure S5. Differential expression analysis of selected genes PD-L1 (CD274), CTLA4 (CD152), LAG3 (CD223), TIGIT (CD155), CD8 and IFNG between WT (n = 6838) and mutated groups in the pan-cancer TCGA cohort. **A.** CREBBP/EP300-MUT-only (n = 487). **B.** POLE/POLD1-MUT-only (n = 209). **C.** CoMut (n = 94). Statistical differences were assessed using the Mann–Whitney test with Bonferroni adjustment. Red denotes mutation groups; grey denotes WT.

A heatmap summarizing the average expression of an expanded panel of immune-related genes across molecular groups is shown in Supplementary Figure S6.

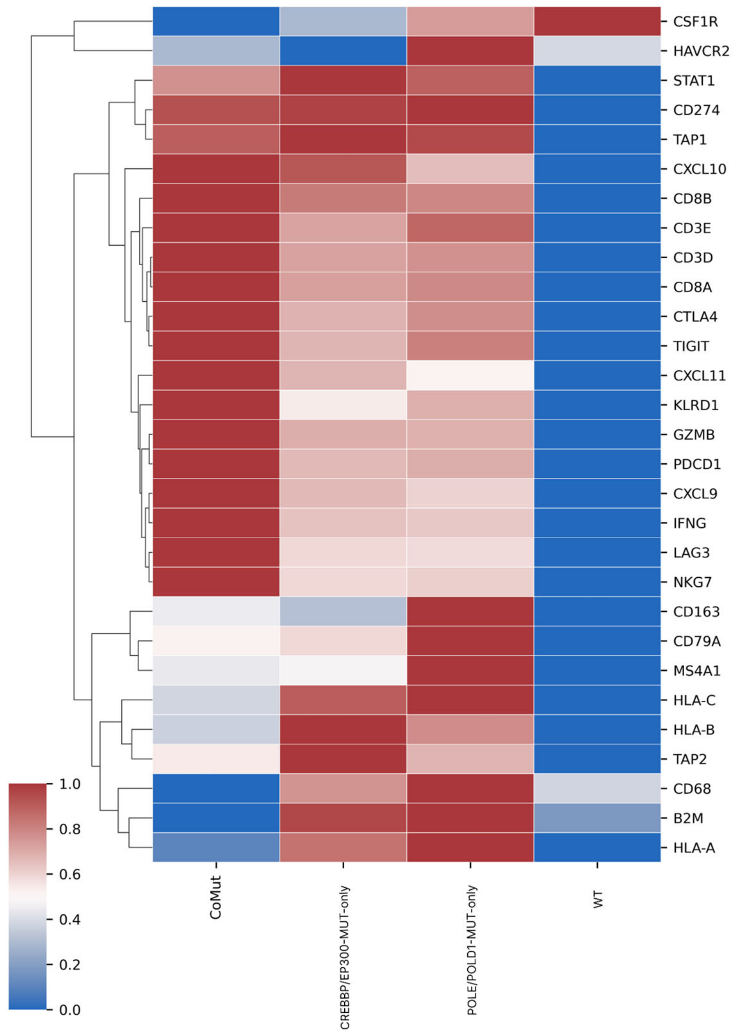

Supplementary Figure S6. Heatmap showing mean expression of immune-related genes across molecular groups. Values represent $\log_2(TPM+0.001)$ averaged per group and scaled per gene (row-wise min–max normalization). Red indicates the highest relative expression among groups, and blue indicates the lowest.

As shown in Supplementary Figure S6, the mutational groups exhibited a consistent upregulation of genes indicative of cytotoxic T-cell infiltration, IFN-γ signature activation, enhanced immune checkpoint expression, and activation of antigen presentation pathways. Interestingly, the CoMut group displayed a relative downregulation of genes involved in antigen presentation compared to the CREBBP/EP300-MUT-only and POLE/POLD1-MUT-only groups.
